# Admission Inflammation Markers Influence Long‐term Mortality in Elderly Patients Undergoing Hip Fracture Surgery: A Retrospective Cohort Study

**DOI:** 10.1111/os.13932

**Published:** 2023-11-20

**Authors:** Anhua Long, Dongxiang Yang, Lu Jin, Feifei Zhao, Xuefei Wang, Yakui Zhang, Liang Liu

**Affiliations:** ^1^ Department of Orthopaedics Beijing Luhe Hospital Affiliated to Capital Medical University Beijing China; ^2^ Evidence‐Based Medicine Center Beijing Luhe Hospital Affiliated to Capital Medical University Beijing China

**Keywords:** C‐reactive protein to albumin ratio, Hip fracture, Inflammation, Long‐term mortality, Neutrophil to lymphocyte ratio

## Abstract

**Objectives:**

Hip fractures in elderly patients are associated with a high mortality rate. Most deaths associated with hip fracture result from complications after surgery. Recent studies suggest that some inflammation biomarkers may be useful to estimate excess mortality. This study aimed to investigate the prognostic value of admission inflammation biomarkers in elderly patients with hip fracture.

**Methods:**

We reports on a retrospective study of elderly hip fracture patients admitted to a hospital in China between January 2015 and December 2019. A total of 1085 patients were included in the study, and their demographic and pre‐operative characteristics were analyzed. The inflammation biomarkers included monocyte to lymphocyte ratio (MLR), neutrophil to lymphocyte ratio (NLR), and C‐reactive protein (CRP) to albumin ratio (CAR). The predictive performance of NLR, MLR and CAR was assessed by receiver operating characteristics (ROC) curve analysis and the association between admission inflammation markers and mortality was evaluated by Cox proportional regression.

**Results:**

The 30‐day, 1‐year, 2‐year, and 4‐year mortality were 1.6%, 11.5%, 21.4% and 48.9%, respectively. The optimal cut‐off values of admission NLR, MLR and CAR for 1‐year mortality were 7.28, 0.76, and 1.36. After adjusting the covariates, preoperative NLR ≥ 7.28 (HR = 1.419, 95% CI: 1.080–1.864, *p* = 0.012) were found to be only independent risk factors with 4‐year all‐cause mortality, the preoperative CAR ≥ 1.36 was independently associated with 1‐year (HR = 1.700, 95% CI: 1.173–2.465, *p* = 0.005), 2 year (HR = 1.464, 95% CI: 1.107–1.936, *p* = 0.008), and 4‐year (HR = 1.341, 95% CI: 1.057–1.700, *p* = 0.016) all‐cause mortality, While age, CCI score, and low hemoglobin at admission were also risk factors for postoperative all‐cause mortality.

**Conclusion:**

Admission CAR and NLR may be useful indicators for predicting the long‐term mortality of elderly patients undergoing hip fracture surgery, and that more research is needed to validate these findings.

## Graphical abstract

Hip fractures in elderly patients are associated with a high mortality rate. Recent studies suggest that some inflammation biomarkers may be useful to estimate excess mortality. This study aimed to investigate the prognostic value of admission inflammation biomarkers in elderly patients with hip fracture. This was a retrospective study of 1085 elderly hip fracture patients. The inflammation biomarkers included monocyte to lymphocyte ratio (MLR), neutrophil to lymphocyte ratio (NLR), and C‐reactive protein (CRP) to albumin ratio (CAR). The predictive performance of NLR, MLR and CAR was assessed by ROC curve analysis and the association between admission inflammation markers and mortality was evaluated by Cox proportional regression. The 30‐day, 1‐year, 2‐year, and 4‐year mortality were 1.6%, 11.5%, 21.4% and 48.9%. After adjusting the covariates, preoperative CAR ≥ 1.36 was associated with 1 year mortality, preoperative NLR ≥ 7.28 and CAR ≥ 1.36 were found to be associated with 4 year mortality. Age, CCI score, and low hemoglobin at admission were also risk factors for postoperative mortality. Admission NLR and CAR may be useful indicators for predicting the long‐term mortality of elderly patients undergoing hip fracture surgery.

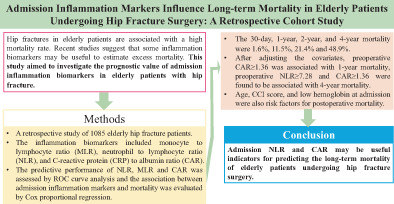

## Introduction

Hip fracture in older people is a global problem that impairs health‐related quality of life and places a substantial socioeconomic burden on the health care system. It is estimated that the number of hip fractures around the world will rise to 6.26 million till 2050.^1^ Increased mortality has been widely reported ranging from 8.4% to 36% during the first year.^2^

Preoperative identification of risk factors associated with high mortality and morbidity will be beneficial for improving prognosis through comprehensive assessment and appropriate management. What is more, comprehensive assessment of the prognosis‐related factors can inform patients about the prognosis. The most reported risk factors associated with mortality are advanced age, male gender, comorbidities, high American Society of Anesthesiologists (ASA) score, time to surgery, cognitive impairment, etc.

Fracture patients suffered with pain, bleeding, immobility, and activated inflammatory, hypercoagulable, and stress states that can precipitate medical complications. The inflammatory pathways are shown to be involved in different conditions in the organism. The bone healing is highly dependent on the inflammatory responses to the injurious stimulus. Neutrophils, monocytes, lymphocytes, and C‐reactive protein (CRP) reflect a systemically inflammatory response and are factors that contribute to bone healing and stress recovery. Whereas, lymphocytes and albumin also reflect the immune and nutritional state of the patients which have been demonstrated as risk factors for mortality in hip fracture. Various preoperative immune‐inflammation index, such as monocyte to lymphocyte ratio (MLR), neutrophil to lymphocyte ratio (NLR), and CRP to albumin ratio (CAR) have been reported to be associated with poor all‐cause mortality in elderly patients undergoing surgery.^3^, ^4^, ^5^, ^6^, ^7^, ^8^ Kim *et al*. reported that preoperative CAR value is related to a higher risk of delirium and death after hip fracture surgery.^9^ A meta‐analysis reported that higher preoperative NLR was correlated with a two folds higher 1‐year mortality among hip fracture patients.^10^ However, certain studies have found no significant association between NLR/MLR and postoperative mortality.^11^, ^12^ Clinical evidence on the association between the preoperative systemic inflammation index and hip fracture mortality in elderly patients remains varied, awaiting further evidence. Few studies have ever attempted to examine the association of these inflammation markers with long‐term mortality beyond 1‐year in hip fracture patients.

The present study, therefore, aimed to investigate whether NRL/MRL/CAR in elderly hip fracture patients undergoing surgery is associated long‐term (1, 2, 4 year) all cause mortality. Furthermore, this study also compared the predictive value of these inflammation markers and the mortality in elderly hip fracture patients.

## Methods

### Study Population

All patients admitted to our hospital between January 2015 and December 2019 with hip fractures were included in the Hip Fracture Database. Patients who fulfilled the inclusion criteria were included in this study. Inclusion criteria: (i) diagnosis of femoral neck or intertrochanteric fracture on admission according to the International Classification of Diseases, 9th edition [ICD‐9]; (ii) age ≥ 60 years; (iii) fresh fracture within 3 weeks; and (iv) low‐energy fracture (a fall from no greater than standing height). The exclusion criteria were: (i) patients with no recording data of routine blood test or CRP or albumin; (ii) with no follow‐up information; (iii) patients diagnosed with chronic or acute infection within 48 h, and (iv) pathological fracture. We also excluded the patients who received conservative therapy. Demographic data (age, gender, race), clinical information (fracture type, admission date, surgery date, the type of surgery, anesthesia type, discharge date, length of stay, postoperative complications, diagnosis), previous history (diabetes, hypertension, coronary heart disease, etc.), American Society of Anesthesiologists (ASA) score, and laboratory data at admission (routine blood test, liver and kidney function) were collected from the above mentioned database by one researcher. Another researcher verified the accuracy of the collected data. Charlson comorbidity index (CCI) score was calculated based on their impact on mortality according to previous report. In this study, CCI was categorized as none (CCI = 0), low (CCI = 1), or high (CCI ≥ 2). This study was conducted in compliance with the World Medical Association Declaration of Helsinki and was approved by the hospital (2021‐LHKY‐127‐01).

### Measurement and Assessment of Inflammation‐related Markers

Whole blood samples (4.0 mL) were routinely obtained from all patients within 24 h after admission, and routine blood test was automatically analyzed with DxH800 Coulter Cellular Analysis System (UniCel DxH800, Beckmann, Indianapolis, IN, USA). CRP was quantified by latex agglutination test and the serum albumin level was determined using bromocresol green method (AU5800, Beckmann). NLR was calculated as the neutrophil counts (normal reference range 1.8–6.3 × 10^9^/L) divided by the lymphocyte counts (normal reference range 1.1–3.2 × 10^9^/L). MLR was calculated as the monocyte counts (normal reference range 0.1–0.6 × 10^9^/L) divided by the lymphocyte counts. CAR was calculated as the CRP level (normal reference range <10 mg/L) divided by the albumin level (normal reference range 40‐55g/L).

### Follow‐up and Outcome

The survival statuses of the patients were telephonically followed up by well‐trained researchers 30 days after discharge, then were followed up routinely every 3 months in the first year, and then every 6 months thereafter until death or the study cut‐off date of March 31, 2022. If the patients were not alive, we should recorded the date and the course of death. If the patients were dead after surgery in hospital, we collected the date of death from medical records. Survival time was calculated from the date of admission to either the date of death or the last follow‐up date (March 31, 2022). The primary outcomes of interest were all‐cause mortality at 1 year.

### Statistical Analysis

All data were exported from the database to WPS tables for variable sorting and data cleaning, and then imported to SPSS software (SPSS 26.0, IBM, Armonk, NY, USA) for statistical analysis. Continuous data were expressed as means (standard deviation, SD) or median (interquartile range, IQR) according to the distribution, and categorical data were described as frequencies (percentages). When comparing data between groups, a chi square test was used for categorical data, student's *t*‐test was used for normally distributed variables, and Wilcoxon rank‐sum test was used for non‐normally distributed variables. The predictive performance of NLR, MLR and CAR was assessed by receiver operating characteristics (ROC) curve analysis and the optimal cut‐off value for prediction of mortality was determined using the Youden index. The Kaplan–Meier method was used for postoperative survival analysis, and a log rank test was used to compare the difference between the survival curves of the groups. Multivariate Cox regression analysis was used to calculate the hazard ratio and 95% confidence interval (95% CI) to determine the independent factors related to the overall mortality. *p* < 0.05 indicated that the difference was statistically significant.

## Results

### The Demographic and Pre‐operative Characteristics of the Study Population

**FIGURE 1:**
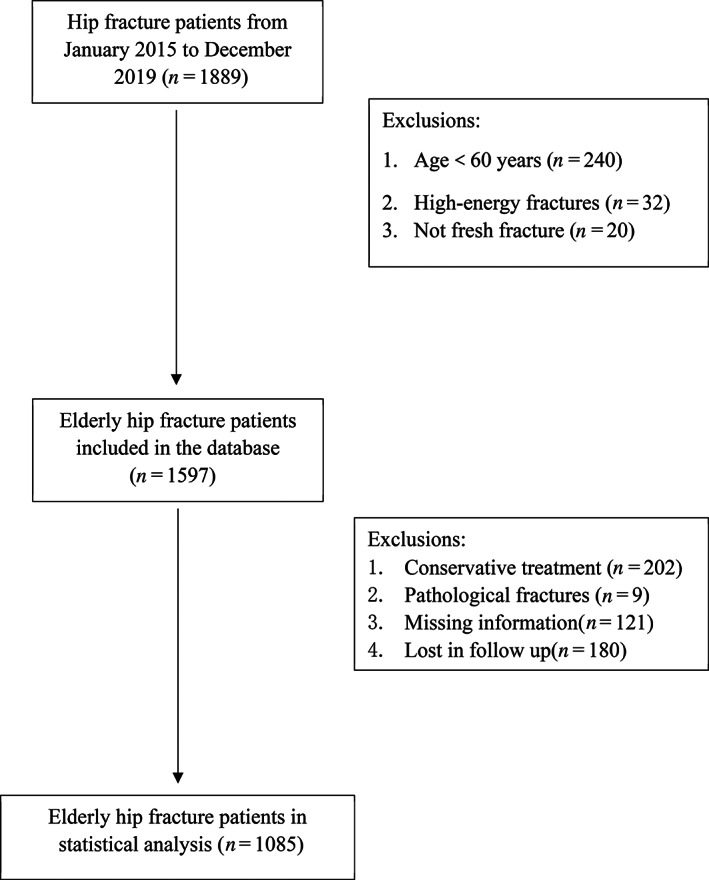
Flowchart of included patients and data analysis in this study.

In total, 1889 hip fracture patients were admitted to our hospital between January 2015 and December 2019. Of these, 804 patients were excluded from the analysis: 240 patients were younger than 60 years old, 32 patients sustained high‐energy trauma, 20 patients were non fresh fracture, nine patients were pathological fractures, 202 patients were treated conservatively, 180 patients were lost during follow up, 121 patients had no recording values for routine blood test or CRP or albumin. Finally, 1085 patients fulfilled the criteria of inclusion and exclusion in this study. Figure 1 shows the flowchart of included patients. The demographic and pre‐operative characteristics were summarized in Table 1. The median (IQR) age was 78 years (71, 84), with 364 males and 721 females, 417 (38.4%) patients were diagnosed with femoral neck fracture and 668 (61.6%) patients were intertrochanteric fracture. Six hundred sixty‐four (61.2%) patients underwent intramedullary fixation surgery and the median (IQR) time to surgery was 5 (3–7) days. Four hundred fifty‐four (41.8%) patients had no comorbidity, while 228 (21.0%) patients were categorized as high comorbidity with CCI ≥ 2. Hypertension (59.8%) was the most common comorbidity, type 2 diabetes (28.7%), prior stroke (22.3%), coronary heart disease (16.0%) followed. Moreover, the medians (IQR) of NLR, MLR, CAR, LCR were 5.68 (3.96–8.62), 0.55 (0.40–0.76), 0.81 (0.33–1.64), 0.04 (0.02–0.09) respectively.

**TABLE 1:** Baseline demographic and pre‐operative characteristics.

Characteristics	Total (*n* = 1085)	Survivors (*n* = 960)	Deaths (*n* = 125)	*p* value
Age (years), median (IQR)	78 (71–84)	77 (70–83)	82 (77–87)	0.000
Gender (male, %)	364 (33.5%)	317 (33.0%)	47 (37.6%)	0.308
Fracture type, *n* (%)				
Femoral neck fracture	417 (38.4%)	372 (38.8%)	45 (36%)	0.836
Intertrochanteric fracture	668 (61.6%)	588 (61.2%)	80 (64%)	
Type of surgery, *n* (%)				
Intramedullary fixation	664 (61.2%)	585 (60.9%)	79 (63.2%)	0.781
Arthroplasty	390 (35.9%)	347 (36.1%)	43 (34.4%)	
Dynamic hip screw or cannulated screw fixation	31 (2.9%)	28 (2.9%)	3 (2.4%)	
Type of anesthesia, *n* (%)				
General anesthesia	122 (11.2%)	110 (11.5%)	12 (9.6%)	0.756
Neuraxial anesthesia	963 (88.8%)	850 (88.5%)	113 (90.4%)	
Hb at admission (g/L)	116.5 ± 18.4	117.5	109.4	0.000
Time to surgery (days), median (IQR)	5 (3–7)	4 (3–7)	5 (3–8)	0.039
CCI score, *n* (%)				
CCI = 0–1	857 (79.0%)	767 (79.9%)	90 (72.0%)	0.047
CCI ≥ 2	228 (21.0%)	193 (20.1%)	35 (28.0%)	
Type 2 diabetes, *n* (%)	311 (28.7%)	278 (29.0%)	33 (26.4%)	0.600
Hypertension, *n* (%)	649 (59.8%)	578 (60.2%)	71 (56.8%)	0.498
Coronary heart disease, *n* (%)	174 (16.0%)	150 (15.6%)	24 (19.2%)	0.364
Prior myocardial infarction, *n* (%)	18 (1.7%)	13 (1.4%)	5 (4.0%)	0.047
Congestive heart failure, *n* (%)	26 (2.4%)	21 (2.2%)	5 (4.0%)	0.211
Chronic respiratory disease, *n* (%)	34 (3.1%)	27 (2.8%)	7 (5.6%)	0.101
Prior stoke, *n* (%)	242 (22.3%)	202 (21.0%)	40 (32.0%)	0.006
Chronic kidney disease, *n* (%)	46 (4.2%)	38 (4.0%)	8 (6.4%)	0.233
Tumor, *n* (%)	50 (4.6%)	48 (5.0%)	2 (1.6%)	0.088
Neutrophil (×10^9^/L), median (IQR)	6.66 (5.23–8.21)	6.66 (5.25–8.20)	6.74 (5.26–8.49)	0.692
Lymphocyte (×10^9^/L), median (IQR)	1.12 (0.80–1.47)	1.13 (0.82–1.48)	1.02 (0.72–1.34)	0.005
Monocyte (×10^9^/L), median (IQR)	0.64 (0.49–0.81)	0.63 (0.49–0.81)	0.68 (0.49–0.91)	0.210
CRP (mg/L), median (IQR)	30.84 (12.63–60.79)	29.22 (12.16–58.53)	45.73 (19.98–89.73)	0.000
Albumin (g/L), median (IQR)	38.6 (36.5–40.7)	38.6 (36.7–40.8)	37.0 (34.4–39.5)	0.000
NLR	5.68 (3.96–8.62)	5.68 (3.89–8.55)	6.19 (4.50–9.38)	0.022
MLR	0.55 (0.40–0.76)	0.54 (0.40–0.74)	0.64 (0.46–0.87)	0.000
CAR	0.81 (0.33–1.64)	0.76 (0.31–1.54)	1.34 (0.50–2.47)	0.000

Abbreviations: CAR, C‐reactive protein to albumin ratio; CCI, Charlson comorbidity index; CRP, C‐reactive protein; IQR, interquartile range; MLR, monocyte to lymphocyte ratio; NLR, neutrophil to lymphocyte ratio.

### The Post‐operative Complications and Mortality of the Study Population

The median (IQR) length of hospital stay was 10 (7.5–14) days. The incidence of postoperative ICU admission was 7.6%, 113 (10.4%) patients had complications. The top five complications were pneumonia (3.8%), thrombosis (3.0%), stoke (2.9%), respiratory failure (2.0%) and heart failure (1.8%). One thousand eighty‐five patients were followed up to 1 year, 1061 patients were followed up to 2 years, and 661 patients were followed up to 4 years. The 30‐day mortality, 1‐year mortality, 2‐year mortality, and 4‐year mortality were 1.6%, 11.5%, 21.4% and 48.9%, respectively (Table 2).

**TABLE 2:** Characteristics of postoperative complications and mortality.

Characteristics	Total (*n* = 1085)
Length of hospital stay (days)	10 (7.5–14)
Postoperative ICU admission, *n* (%)	82 (7.6%)
Complication, *n* (%)	113 (10.4%)
Incision infection, *n* (%)	1 (0.1%)
Pneumonia, *n* (%)	41 (3.8%)
Urinary tract infection, *n* (%)	14 (1.3%)
Respiratory failure, *n* (%)	22 (2.0%)
Gastrointestinal bleeding, *n* (%)	4 (0.4%)
Thrombosis, *n* (%)	33 (3.0%)
Circulatory system complication, *n* (%)	39 (3.6%)
Heart failure, *n* (%)	20 (1.8%)
Myocardial infarction, *n* (%)	18 (1.7%)
Angina, *n* (%)	8 (0.7%)
Arrhythmia, *n* (%)	14 (1.3%)
Stroke, *n* (%)	31 (2.9%)
30‐day mortality	14/1085 (1.6%)
1‐year mortality	125/1085 (11.5%)
2‐year mortality	227/1061 (21.4%)
4‐year mortality	324/662 (48.9%)

### ROC Curve Analysis

Cut‐off values of admission NLR, MLR and CAR for 1‐year mortality were evaluated by ROC analysis. The optimal cut‐off value of NLR was 7.28 with sensitivity of 45.6% and specificity of 66.4% (area under the curve: 0.57; 95% CI: 0.510–0.616; *p* = 0.013; Table 3). For MLR, the optimal cut‐off value was 0.76 with sensitivity of 41.2% and specificity of 76.9% (area under the curve: 0.61; 95% CI: 0.551–0.658; *p* = 0.000; Table 3). For CAR, the optimal cut‐off value was 1.36 with sensitivity of 49.6% and specificity of 71.4% (area under the curve: 0.63; 95% CI: 0.576–0.686; *p* = 0.000; Table 3).

**TABLE 3:** ROC results of NLR, MLR and CAR.

Statistical analysis	NLR	MLR	CAR
Cut‐off value	7.28	0.76	1.36
AUC	0.57	0.61	0.63
*p* value	0.013	0.000	0.000
Sensitivity	0.456	0.412	0.496
Specificity	0.664	0.769	0.714
95%CI	0.510–0.616	0.551–0.658	0.576–0.686

Abbreviations: AUC, area under the curve; CAR, C‐reactive protein to albumin ratio; MLR, monocyte to lymphocyte ratio; NLR, neutrophil‐to‐lymphocyte ratio; ROC, receiver operating characteristic curve.

### Kaplan–Meier Analysis

**FIGURE 2:**
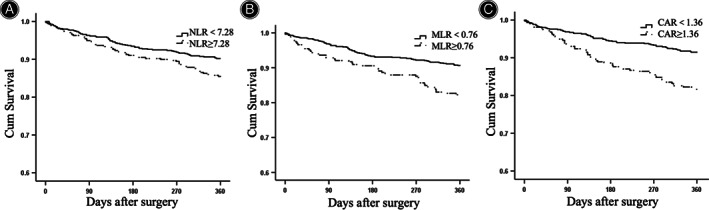
Kaplan–Meier analysis of 1‐year survival after surgery. (A) *Cum* survival of the subjects in neutrophil to lymphocyte ratio (NLR) < 7.28 group versus NLR ≥ 7.28 group. (B) *Cum* survival of the subjects in monocyte to lymphocyte ratio (MLR) < 0.76 group versus MLR ≥ 0.76 group. (C) *Cum* survival of the subjects in C‐reactive protein (CAR) < 1.36 group versus CAR ≥ 1.36 group.

We divided the subjects into two groups according to the cut‐off values of admission NLR, MLR and CAR values and survival analysis was evaluated between the groups. For 1‐year analysis, the survival rate of NLR < 7.28 group was significantly higher than NLR ≥ 7.28 group by log rank test (90.2% *vs*. 85.2%, *p* = 0.015) (Figure 2A). The survival rate of MLR < 0.76 group was 90.5%, while the survival rate of MLR ≥ 0.76 group was 82.3%, the difference was significant with a *p* value of 0.000 (Figure 2B). Also, the survival rate of CAR < 1.36 group was significantly higher than CAR ≥ 1.36 group (91.3% *vs*. 81.6%, *p* = 0.000) (Figure 2C). 2‐year survival analysis showed that the survival rates were significantly higher in lower NLR group *vs*. higher NLR group (81.5% *vs*. 73.2%, *p* = 0.002) (Figure S2A). The survival rates were 80.8% *vs*. 71.9% (*p* = 0.002) for lower MLR group and higher MLR group (Figure S2B). For the groups divided by CAR, the survival rates were 82.4% *vs*. 70.4% (*p* = 0.000) (Figure S2C). Similar results were obtained for 4‐year survival analysis (Figure S2A–C).

### Univariate Cox Hazardous Proportional Analysis for the Risk Factors Associated with Mortality

As shown in Table 4, univariate analysis showed that increasing age (HR = 1.073, 95% CI: 1.049–1.096, *p* = 0.000), high CCI score (HR = 1.179, 95% CI: 1.001–1.389, *p* = 0.049), hemoglobin at admission (HR = 0.978, 95% CI: 0.969–0.987, *p* = 0.000), NLR ≥ 7.28 (HR = 1.542, 95% CI: 1.084–2.194, *p* = 0.016), MLR ≥ 0.76 (HR = 1.948, 95% CI: 1.356–2.798, *p* = 0.000), and CAR ≥ 1.36 (HR = 2.234, 95% CI: 1.559–3.200, *p* = 0.000) were risk factors of 1‐year mortality. The higher the hemoglobin at admission, the lower risk of death. Similar results were found for 2‐year mortality and 4‐year mortality (Table 4). Femoral neck fracture was significantly associated with 2‐year mortality (HR = 0.751, 95% CI: 0.569–0.991, *p* = 0.043) and 4‐year mortality (HR = 0.768, 95% CI: 0.611–0.966, *p* = 0.024) (Table 4). Set Intramedullary fixation as reference, arthroplasty was associated with 2‐year mortality (HR = 0.745, 95% CI: 0.561–0.991, *p* = 0.043). Gender, fracture type, type of anesthesia, type of surgery, and time to surgery were not found to be associated with mortality (Table 4).

**TABLE 4:** Univariate cox regression analysis for the risk factors associated with 1‐year mortality, 2‐year mortality and 4‐year mortality.

Characteristics	1‐year mortality	*p*	2‐year mortality	*p*	4‐year mortality	*p*
	HR (95% CI)		HR (95% CI)		HR (95% CI)	
Age	1.073 (1.049–1.096)	0.000	1.058 (1.041–1.075)	0.000	1.057 (1.042–1.071)	0.000
Female	0.842 (0.586–1.209)	0.351	0.927 (0.706–1.217)	0.586	0.918 (0.732–1.152)	0.461
CCI	1.179 (1.001–1.389)	0.049	1.188 (1.052–1.341)	0.005	1.172 (1.057–1.301)	0.003
Femoral neck fracture	0.888 (0.616–1.279)	0.523	0.751 (0.569–0.991)	0.043	0.768 (0.611–0.966)	0.024
Neuraxial anesthesia	1.205 (0.665–2.185)	0.539	0.924 (0.618–1.380)	0.698	1.064 (0.752–1.505)	0.725
Type of surgery						
Intramedullary fixation	Reference		Reference		Reference	
Arthroplasty	0.913 (0.630–1.324)	0.632	0.745 (0.561–0.991)	0.043	0.795 (0.630–1.002)	0.795
Dynamic hip screw or cannulated screw fixation	0.827 (0.261–2.619)	0.747	0.854 (0.378–1.931)	0.705	0.563 (0.250–1.268)	0.166
Hb at admission	0.978 (0.969–0.987)	0.000	0.978 (0.971–0.984)	0.000	0.982 (0.976–0.987)	0.000
Time to surgery	1.000 (0.994–1.006)	0.949	0.999 (0.993–1.005)	0.827	0.998 (0.992–1.004)	0.478
NLR ≥ 7.28	1.542 (1.084–2.194)	0.016	1.520 (1.170–1.976)	0.002	1.502 (1.207–1.869)	0.000
MLR ≥ 0.76	1.948 (1.356–2.798)	0.000	1.563 (1.183–2.066)	0.002	1.469 (1.165–1.852)	0.001
CAR ≥ 1.36	2.234 (1.559–3.200)	0.000	1.840 (1.403–2.413)	0.000	1.659 (1.318–2.089)	0.000

Abbreviations: CAR, C‐reactive protein to albumin ratio; CCI, Charlson comorbidity index; CI, confidence interval; MLR, monocyte to lymphocyte ratio; NLR, neutrophil‐to‐lymphocyte ratio.

### Multivariate Cox Hazardous Proportional Analysis for the Risk Factors Associated with Mortality

Risk factors of age, gender, CCI, fracture type, type of anesthesia, type of surgery, time to surgery, hemoglobin at admission and the inflammatory factors (NLR, MLR, and CAR) were included in multivariate Cox regression analysis. After adjusting the covariates, only Preoperative CAR ≥ 1.36 was independently associated with 1‐year mortality (HR = 1.700, 95% CI: 1.173–2.465, *p* = 0.005) and 2‐year mortality (HR = 1.464, 95% CI: 1.107–1.936, *p* = 0.008) (Table 5). For 4‐year mortality, both NLR ≥ 7.28 (HR = 1.419, 95% CI: 1.080–1.864, *p* = 0.012) and CAR ≥ 1.36 (HR = 1.341, 95% CI: 1.057–1.700, *p* = 0.016) were found to be independent risk factors (Table 5). Furthermore, increasing age, high CCI, and hemoglobin at admission were significantly associated with 1‐year mortality, 2‐year mortality, and 4‐year mortality as well (Table 5). Gender, type of anesthesia, type of surgery, time to surgery, and MLR ≥ 0.76 were not found to be associated with mortality (Table 5).

**TABLE 5:** Multivariate Cox regression analysis for the risk factors associated with 1‐year mortality, 2‐year mortality and 4‐year mortality.

Characteristics	1‐year mortality	*p*	2‐year mortality	*p*	4‐year mortality	*p*
	HR (95% CI)		HR (95% CI)		HR (95% CI)	
Age	1.060 (1.034–1.086)	0.000	1.045 (1.026–1.064)	0.000	1.043 (1.027–1.059)	0.000
Female	0.696 (0.473–1.025)	0.067	0.748 (0.558–1.004)	0.053	0.853 (0.668–1.089)	0.202
CCI	1.159 (0.983–1.367)	0.079	1.187 (1.050–1.343)	0.006	1.195 (1.076–1.326)	0.001
Femoral neck fracture	0.865 (0.207–3.612)	0.843	0.893 (0.272–2.931)	0.852	0.724 (0.258–2.032)	0.539
Neuraxial anesthesia	1.147 (0.615–2.140)	0.665	0.967 (0.626–1.494)	0.881	0.961 (0.664–1.391)	0.834
Type of surgery						
Intramedullary fixation	Reference		Reference		Reference	
Arthroplasty	1.617 (0.385–6.790)	0.512	1.224 (0.369–4.060)	0.741	1.580 (0.562–4.447)	0.386
Dynamic hip screw or cannulated screw fixation	2.245 (0.369–13.655)	0.380	2.210 (0.541–9.025)	0.269	1.767 (0.467–6.682)	0.401
Hb at admission	0.984 (0.973–0.994)	0.002	0.982 (0.974–0.990)	0.000	0.986 (0.979–0.993)	0.000
Time to surgery	1.001 (0.994–1.008)	0.795	1.000 (0.994–1.007)	0.940	0.998 (0.991–1.005)	0.585
NLR ≥ 7.28	1.173 (0.759–1.813)	0.472	1.374 (0.991–1.905)	0.056	1.419 (1.080–1.864)	0.012
MLR ≥ 0.76	1.369 (0.863–2.171)	0.182	1.041 (0.730–1.484)	0.825	0.983 (0.729–1.325)	0.909
CAR ≥ 1.36	1.700 (1.173–2.465)	0.005	1.464 (1.107–1.936)	0.008	1.341 (1.057–1.700)	0.016

Abbreviations: CAR, C‐reactive protein to albumin ratio; CCI, Charlson comorbidity index; CI, confidence interval; MLR, monocyte to lymphocyte ratio; NLR, neutrophil‐to‐lymphocyte ratio.

## Discussion

In this study, we discovered a significant correlation between CAR and all‐cause mortality at 1, 2, 4 years after hip fracture surgery. Additionally, we found that NLR was only associated with all‐cause mortality at the 4‐year mark. However, admission MLR was not an independent predictor of death compared to CAR and NLR. It means that admission CAR and NLR might be useful biomarkers for predicting long‐term mortality in hip fracture patients undergoing surgery.

## Inflammation Predict Long‐term Mortality after Hip Fracture Surgery

The overall mortality rates of elderly hip fracture patients at 30 days, 1, 2 and 4 years were 1.6%, 11.5%, 21.4% and 48.9%. In 2021, Wang *et al*.^13^ reported that the postoperative mortality rates at 30 days and 1 year were 4.5% and 9.0%, respectively. Previous studies reported that 30‐day mortality rate ranged from 1.4% to 10%, and this rate decreased year by year.^14^, ^15^ In our study, the 30‐day mortality rate was relatively low, which might be explained by the fact that in China patients with poor overall status are more inclined to conservative treatment and we just calculated the rate in patients under operation. A meta‐analysis calculated the 1‐year mortality rate was 13.96% after hip fracture and this rate increased with age.^16^ A retrospective cohort study reported the 2‐year and 4‐year all‐cause mortality rate was 12.9% and 20.1%, respectively.^17^ However, in our study both the rates were higher. The advanced age might partially explained the disparity.

As we know, neutrophils, lymphocytes, and monocytes are the laboratory parameters in blood routine test, which is a simple, cheap and easily available test. Neutrophils and monocytes are pro‐inflammatory cells of innate immunity, while lymphocytes are effector cells of adaptive immunity. Thus, NLR and MLR are markers conjugated the innate and adaptive immunity, which can comprehensively and accurately reflect the systemic inflammation state. NLR has been established as a good indicator of systemic inflammation in prevalent chronic diseases, such as diabetes, hypertension and coronary heart diseases.^18^, ^19^ Moreover, previous studies have revealed that admission NLR value could be a prognostic factor for 1‐year mortality in elderly patients with hip fracture.^10^, ^20^ And also, MLR has been suggested to be associated with poor survival in elderly patients with hip fracture.^8^, ^21^ The NLR and MLR are additional inflammation‐based markers that have been widely studied in various medical conditions, including cardiovascular diseases and cancer. Both ratios are calculated by dividing the absolute count of neutrophils or monocytes, respectively, by the absolute count of lymphocytes. These ratios serve as surrogate markers of systemic inflammation and have been shown to have prognostic value in different patient populations. In the context of hip fracture patients, previous research on NLR and MLR has demonstrated their association with mortality and other adverse outcomes. The underlying mechanisms explaining these associations are not fully elucidated but may be related to the inflammatory response triggered by the fracture itself, as well as the presence of comorbidities and age‐related changes in immune function. CRP, another inflammation marker, has also been demonstrated as a prognostic factor for mortality in hip fracture patients.^6^, ^22^ Albumin, which reflects the nutrition state, has been suggested to affect the prognosis in elderly patients.^23^ CAR, a marker conjugated the inflammation and nutrition, had been considered as a risk factor for 1‐year mortality in hip fracture patients.^6^, ^24^ Our results revealed that admission CAR was associated with 1, 2, and 4‐year mortality. According to the ROC curve and Youden index, the cut‐off values of NLR, MLR and CAR were set as 7.28, 0.76 and 1.36, respectively. According to the cut‐off values, the patients were divided into two groups. The high values group had higher mortality rate, which demonstrated that the inflammation markers affected the prognosis of patients. In this study, the results of multivariate analysis revealed that admission NLR and CAR could be regarded as significant prognostic factors for long‐term (≥1 year) mortality. MLR was found to be a significant risk factor for mortality in the univariate analysis. However, this significance diminished after adjusting for other risk factors. It means that CAR might be the best immune‐inflammation biomarker for predicting all‐cause long‐term mortality in elderly hip fracture patients.

Furthermore, in our study multivariate Cox regression analysis showed that age, CCI score and hemoglobin level at admission were all risk factors for postoperative death. Advanced age was associated with higher mortality, which was consistent with previous studies.^25^, ^26^ The CCI score is a comprehensive evaluation based on patients' comorbidities. The higher the score, the more types or severity of comorbidities. It was not difficult to understand that the CCI score was a independent risk factor for postoperative mortality, which was consistent with a previous study.^27^ Similar to previous reports,^28^ the higher the hemoglobin at admission, the lower risk of death. And also, previous studies have shown that preoperative correction of anemia will reduce the incidence of postoperative complications and postoperative mortality.^29^ In our study, the male patients accounted for less, that was about 33.5%. Previous studies on hip fracture also showed that the proportion of males was relatively low, about 21%–27%.^30^, ^31^ Whether gender was a risk factor for death remained controversial.^32^ Some studies showed that male patients had a higher risk of death,^33^ while other studies showed the opposite.^32^ However, in our study we found that gender was not a independent risk factor for death.

## Limitations and Prospects

However, this study had some limitations. First, the single‐center study can only represent the patients admitted to our hospital. Second, we did not include all variables associated with the mortality of patients, such as body mass index. Third, in the follow‐up process of this study, the cause of death could not be accurately recorded and further analysis could not be made. Finally, we did not record postoperative laboratory data that we could not analyze whether the changes in these inflammatory markers are associated with the prognosis. Thus, in the future, multi‐center research is needed to expand the sample size so as to draw more comprehensive and clear conclusions. This information can help healthcare providers identify patients who are at higher risk of long‐term mortality and allocate resources and interventions accordingly. Clinicians can use the identified biomarkers as additional factors to consider when making treatment decisions for hip fracture patients. Patients at higher risk based on these biomarkers might benefit from more intensive monitoring, early interventions, or specialized care pathways.

## Conclusion

In conclusion, this study revealed that admission NLR and CAR could be regarded as a significantly prognostic factors for long‐term(≥ 1 year) mortality. Furthermore, Age, CCI score and low hemoglobin at admission were risk factors for postoperative mortality.

## Funding Information

This work was supported by The Capital's Funds for Health Improvement and Research (2020–2‐7081) and The Tongzhou District Science and Technology Commission Project (KJ2020CX004–21).

## Conflict of Interest Statement

All named authors have no conflicts of interest to disclose in relation to this article.

## Ethics Statement

The study was approved by the ethics review board of Beijing Luhe Hospital Affiliated to Capital Medical University (No. 2021‐LHKY‐127‐01) in accordance with the Declaration of Helsinki. Written informed consent was obtained from all individual patients included in the study.

## Author Contributions

Liang Liu and Anhua Long: conceptualization, methodology, software. Anhua Long: data curation, writing—original draft preparation. Dongxiang Yang: visualization, investigation. Feifei Zhao and Yakui Zhang: supervision. Lu Jin: software, validation. Xuefei Wang: writing—reviewing and editing. All authors read and approved the final manuscript.

## Authorship Declaration

All authors listed meet the authorship criteria according to the latest guidelines of the International Committee of Medical Journal Editors, and all authors are in agreement with the manuscript.

## Supporting information


**Figure S1.** Kaplan–Meier analysis of 2‐year survival after surgery. (A) *Cum* survival of the subjects in NLR < 7.28 group versus NLR ≥ 7.28 group. (B) *Cum* survival of the subjects in MLR < 0.76 group versus MLR ≥ 0.76 group. (C) *Cum* survival of the subjects in CAR < 1.36 group versus CAR ≥ 1.36 group.
**Figure S2.** Kaplan–Meier analysis of 4‐year survival after surgery. (A) *Cum* survival of the subjects in NLR < 7.28 group versus NLR ≥ 7.28 group. (B) *Cum* survival of the subjects in MLR < 0.76 group versus MLR ≥ 0.76 group. (C) *Cum* survival of the subjects in CAR < 1.36 group versus CAR ≥ 1.36 group.
