# Supplementary material for: Admission Inflammation Markers Influence Long‐term Mortality in Elderly Patients Undergoing Hip Fracture Surgery: A Retrospective Cohort Study
Source: Orthop Surg. 2023 Nov 20;16(1):38–46. doi: 10.1111/os.13932 (PMC10782247; doi:10.1111/os.13932)
Supplement: Supplementary file 1 — Figure S1. Kaplan–Meier analysis of 2‐year survival after surgery. (A) Cum survival of the subjects in NLR < 7.28 group versus NLR ≥ 7.28 group. (B) Cum survival of the subjects in MLR < 0.76 group versus MLR ≥ 0.76 group. (C) Cum survival of the subjects in CAR < 1.36 group versus CAR ≥ 1.36 group. Figure S2. Kaplan–Meier analysis of 4‐year survival after surgery. (A) Cum survival of the subjects in NLR < 7.28 group versus NLR ≥ 7.28 group. (B) Cum survival of the subjects in MLR < 0.76 group versus MLR ≥ 0.76 group. (C) Cum survival of the subjects in CAR < 1.36 group versus CAR ≥ 1.36 group. [file OS-16-38-s001.docx]

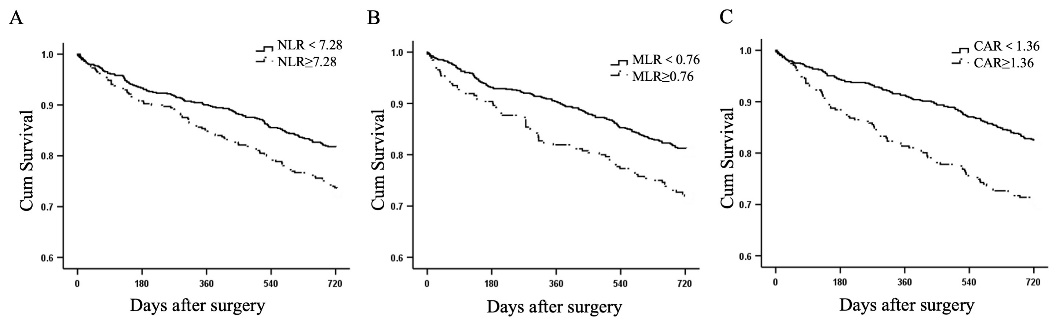


Supplementary Figure 1 Kaplan-Meier analysis of 2-year survival after surgery. (A) Cum survival of the subjects in NLR < 7.28 group vs NLR ≥ 7.28 group. (B) Cum survival of the subjects in MLR < 0.76 group vs MLR ≥ 0.76 group. (C) Cum survival of the subjects in CAR < 1.36 group vs CAR ≥ 1.36 group.


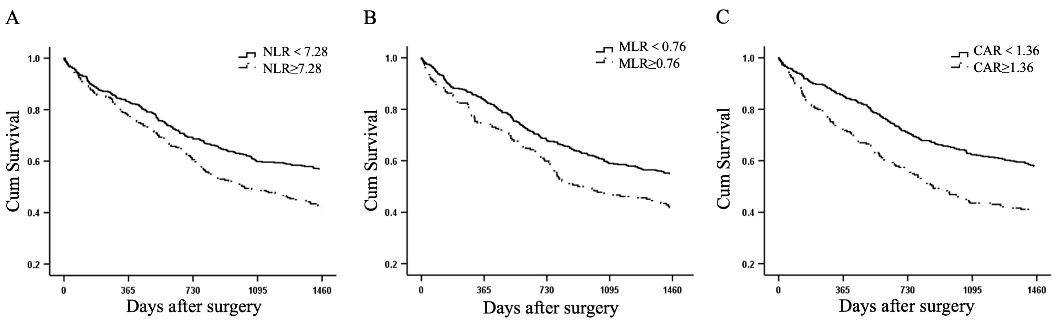


Supplementary Figure 2 Kaplan-Meier analysis of 4-year survival after surgery. (A) Cum survival of the subjects in NLR < 7.28 group vs NLR ≥ 7.28 group. (B) Cum survival of the subjects in MLR < 0.76 group vs MLR ≥ 0.76 group. (C) Cum survival of the subjects in CAR < 1.36 group vs CAR ≥ 1.36 group.
